# Protocol to isolate stress granules in HeLa cells using fluorescence-activated non-membrane condensate isolation

**DOI:** 10.1016/j.xpro.2025.103987

**Published:** 2025-07-26

**Authors:** Yilong Zhou, Maria Shvedunova, Asifa Akhtar

**Affiliations:** 1 Max Planck Institute of Immunobiology and Epigenetics, 79108 Freiburg, Germany; 2 Cancer Science Institute of Singapore, National University of Singapore, Singapore 117599, Singapore

**Keywords:** Cell Biology, Cell culture, Cell isolation, Flow Cytometry, Genetics, Molecular Biology

## Abstract

Various stress stimuli induce cytosolic stress granules (SGs) in mammalian cells, which are composed of RNA and RNA-binding proteins and have important physiological functions. Here, we present a protocol for isolating SGs using fluorescence-activated non-membrane condensate isolation (FANCI). We describe steps for seeding and stressing G3BP1-mCherry HeLa cells. We then detail procedures for purifying SGs by FANCI with flow cytometry. This protocol has potential application in studying SGs from other cells in the future.

For complete details on the use and execution of this protocol, please refer to Zhou et al.^1^

## Highlights

• Administering specific stress treatments to cells to induce SGs
• Instructions for preparing SG-containing cell lysates
• Explanation of the flow cytometry-based SG purification procedure
• Procedures for RNA extraction from purified SGs

## Abstract (editor highlights)

Publisher’s note: Undertaking any experimental protocol requires adherence to local institutional guidelines for laboratory safety and ethics.

## Graphical abstract

**Figure:**
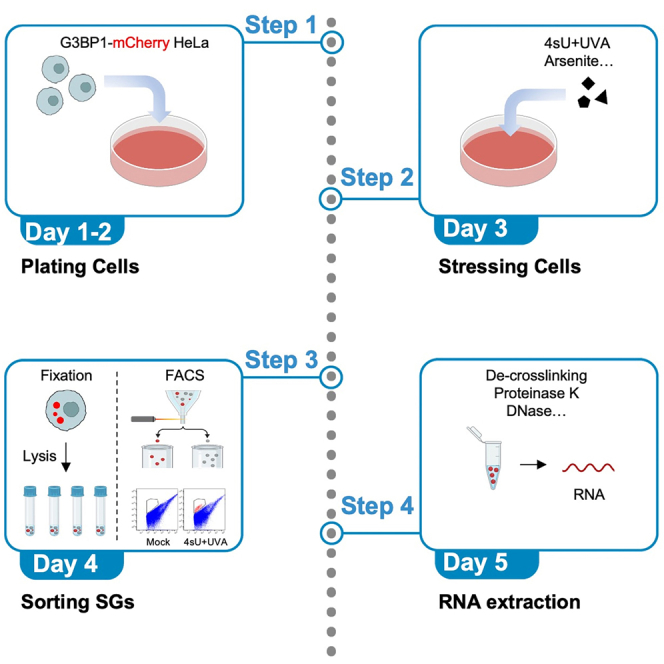

## Before you begin

Stress granules are cytosolic biomolecular condensates that form in response to stress and crosstalk with other stress responses such as autophagy and antiviral immunity.^2^^,^^3^^,^^4^ Proximal labeling and antibody-based methods have been applied to isolate SGs but suffer from low efficiency and high background due to the fact that SGs are tightly associating to endoplasmic reticulum (ER) and nuclear membranes.^1^^,^^5^^,^^6^ We developed the FANCI technology to isolate SGs from cells by flow cytometry with high efficiency and low background.^1^ The protocol below describes the specific steps for using HeLa cells expressing endogenously mCherry-tagged G3BP1 to isolate arsenite-, heat shock-, osmosis- and 4sU+UVA-induced SGs. The protocol has not yet been tested on SGs induced by other kinds of stress. We predict that this protocol will also achieve similar results with other fluorescent tags and other tagged SG proteins.

### Cell line recovery and culture

**Timing: 7 days prior to FANCI protocol**

1. Recovering cryopreserved HeLa cells.
  ***Note:*** Recover G3BP1-mCherry HeLa at least one week before the experiment.
  a. Rapidly thaw the frozen stocks (in liquid nitrogen) of cells at 37°C.
  b. Resuspended in 10 mL of prewarmed (37°C) DMEM media (DMEM high glucose GlutaMAX from Thermo Fisher Scientific).
2. Centrifuge the resuspended cells at 200 rpm for 3 min and discard the supernatant.
3. Resuspend the pelleted cells in 1 mL of prewarmed (37°C) DMEM culturing media (DMEM high glucose GlutaMAX supplemented with 10% (v/v) heat-inactivated fetal bovine serum, 1% (v/v) penicillin–streptomycin).
  a. Transfer the cells to a 10 cm^2^ culture dish (Corning 10 cm^2^ vented cell culture flasks) with 9 mL of prewarmed (37°C) DMEM culturing media.
4. Incubate the culture flask at 37°C with 5% CO_2_. Check cell state under an optical microscope and exchange the culturing media in the culture dish with fresh DMEM culturing media every two days.
5. Split the cultured cells at least three times over one week to ensure the cells will be at optimal health.

***Note:*** Confirm that cells are free of mycoplasma using the Mycoplasma PCR Detection before the experiment.

***Note:*** Cells should be in an optimal state before the experiment, as SGs can form spontaneously under some suboptimal conditions. Additionally, some stress-induced SG formation is highly sensitive to cell state, which could introduce variability in experimental outcomes. The healthy state of HeLa cells can be referenced on the ATCC website (ATCC: HeLa, CCL-2).

***Note:*** The method for generating G3BP1-mCherry HeLa-CCL-2 cells using CRISPR-Cas9 is described in our previous study.^1^ Primers used for the repair template cloning into pcDNA5 (Thermo, V103320) and sgRNA cloning into pX459 (Addgene #62988) are listed in the study.^1^ The repair template plasmid and pX459-sgRNA plasmid targeting the G3BP1 locus were co-transfected at a 3:1 ratio using Lipofectamine 3000 (Thermo Fisher, L3000001) in 6-well plates. After 48 h, cells were selected with 1 μg/ml puromycin for 3 days, then replated into 10 cm dishes for recovery. Single cells were sorted into 96-well plates based on high mCherry fluorescence. Clones were expanded and screened for homozygous G3BP1 tagging by genomic PCR and confirmed by western blot.

## Key resources table

**Table:** 

REAGENT or RESOURCE	SOURCE	IDENTIFIER
**Experimental models: Cell lines**		

G3BP1-mCherry HeLa	Zhou et al.^1^	N/A

**Chemicals, peptides, and recombinant proteins**		

HEPES	Fisher Scientific	BP310-100
PBS	Corning	21-040-CV
KCl	Fisher Scientific	P217-500
NaCl	Fisher Scientific	S640-500
DTT	Sigma	43816
NP40 (Igepal CA-630)	Sigma	I8896
16% formaldehyde	Thermo Fisher Scientific	28908
Glycine	Fisher Scientific	BP381-500
Sodium (meta) arsenite (arsenite, As)	Sigma	s7400
4-thiouridine (4sU)	Sigma	T4509

**Other**		

Dulbecco’s modified Eagle’s medium (DMEM), low glucose GlutaMAX supplement, pyruvate	Thermo Fisher Scientific	10567014
Gibco penicillin-streptomycin (10,000 U/mL)	Gibco/Thermo Fisher Scientific	15140122
Trypsin-EDTA (0.25%), phenol red	Gibco/Thermo Fisher Scientific	25200056
Murine RNase inhibitor	New England Biolabs GmbH	M0314L
TURBO DNase	Thermo Fisher Scientific	AM2238
Proteinase-K	Thermo Fisher Scientific	EO0491
Oligo-clean and concentrator (OCC) kit	Zymo Research	D4060
Protease inhibitor cocktail	Roche	11836170001
PhosSTOP	Roche	4906845001
Cell culture dish (15 cm)	Corning	430599
Cell culture dish (10 cm)	Corning	430167
40 μM filter	Corning	431750
DNA LoBind tubes	Eppendorf	0030108051
Cell scraper 39 cm 30 mm	VWR	734-2605
Bioruptor	Diagenode	B01020001
Flow cytometers: S6	Becton Dickinson (BD)	Symphony S6 sorters
Flow cytometers: FACSAria Fusion	Becton Dickinson (BD)	BD FACSAria Fusion Flow Cytometer
UVA crosslinker	VILBER	Bio-Link, BLX-365

## Materials and equipment

### Materials

**Table:** B0 buffer (40 mL)

Reagents	Final concentration	Stock concentration	Amount
HEPES (pH 7.5)	50 mM	100 mM	20 mL
KCl	150 mM	3 M	2 mL
NP40(IGEPAL CA-630)	1%	N/A	400 μL
Protease Inhibitor Cocktail	N/A	N/A	2 tablets
PhosSTOPPhosphatase Inhibitor Cocktail	N/A	N/A	2 tablets
H_2_O	N/A	N/A	17.6 ml

Make fresh, 40 mL of B0 buffer is sufficient for processing nine samples.

**Table:** Quenching buffer (2.5 M glycine) (200 mL)

Reagents	Final concentration	Stock concentration	Amount
Glycine	2.5 M	N/A	37.5 g
H_2_O	N/A	N/A	200 ml

Quenching buffer can be stored for up to 1 year at 25°C.

**Table:** 2 M NaCl (100 mL)

Reagents	Final concentration	Stock concentration	Amount
NaCl	2 M	N/A	11.7 g
H_2_O	N/A	N/A	100 ml

NaCl solution can be stored for up to 3 years at 25°C.

**Table:** 200 mM arsenite (100 mL)

Reagents	Final concentration	Stock concentration	Amount
sodium arsenite	200 mM	N/A	26.0 mg
H_2_O	N/A	N/A	1 ml

Arsenite solution can be stored for up to 3 years at −20°C.

**Table:** 500 mg/ml 4sU (0.5 mL)

Reagents	Final concentration	Stock concentration	Amount
4sU	500 μg/ml	500 mg/ml	250 mg
H_2_O	N/A	N/A	0.5 ml

It is important to aliquot and store 4sU solutions at −20°C, protect them from light, and avoid repeated thawing and freezing cycles.

### Equipment setup

#### Flow cytometer

The protocol below describes the specific steps using the Becton Dickinson (BD) Symphony S6 sorter. However, we have also successfully conducted the same protocol with the AriaFusion (BD) Flow Cytometer.

The 70 μm nozzle was used for both machines. Forward scatter (FSC) and side scatter (SSC) were settled as 200, which is critical for the protocol as it enables the sorter to capture SGs events from the cell lysate.

#### UV crosslinker

Before starting, confirm that all the lamps in the UVA crosslinker (VILBER, Bio-Link) are UVA type (∼365 nM) and working properly.

**Figure 1:**
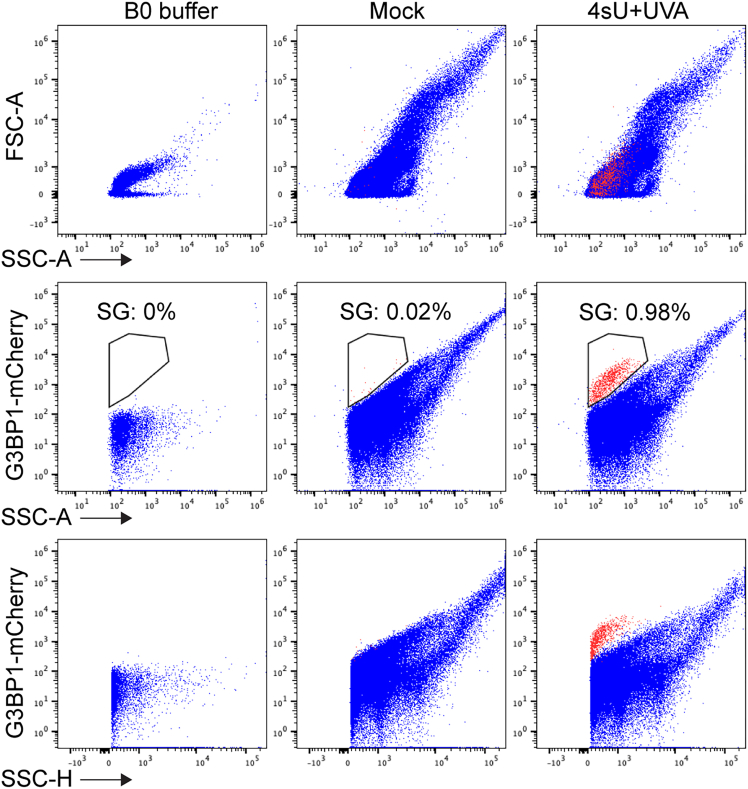
Representative FACS profiles generated from this protocol All the channels must be in logarithmic view. For SG gating, display all events in a graph [y-axis = mCherry; x-axis = SSC-A]. Draw gate around the population of SGs which have a higher mCherry signal intensity as shown in middle panel. Red indicates gated SGs and blue indicates all other particles in the cell lysates.

**Figure 2:**
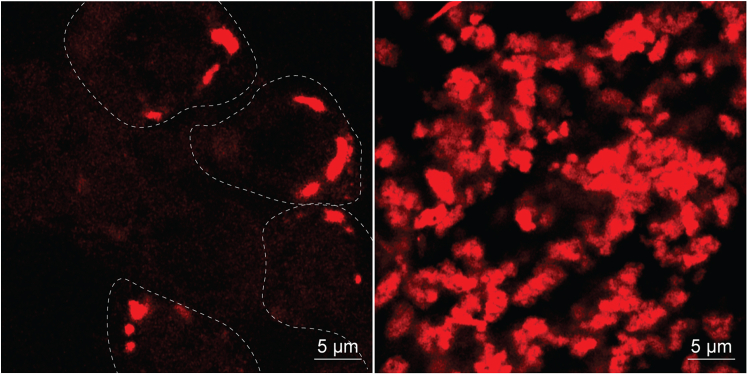
Representative images generated from this protocol 4sU+UVA-induced SGs in cells (left panel) and isolated by FANCI (right panel).] White dash line marks the SG-containing cells. Scale bars, 5 μm.

## Step-by-step method details

### Cell plating and treatment

**Timing: 3 days**

**Timing: 8 h prior to fixation (for step 2)**

1. Plating cells in 15 cm dishes.
  a. Aspirate and discard the cell culturing medium from a 10 cm^2^ culture dish of G3BP1-mCherry HeLa cells. Wash the cells once with 1 ml Trypsin-EDTA.
  b. Add 1 mL Trypsin-EDTA, gently shake the dish, let the trypsin cover the cells.
  c. Put the dish in the 37°C incubator for 3 min.
  d. Add 10 mL prewarmed DMEM culturing media to stop the trypsin-EDTA reaction.
  e. Collect all the liquid in a new 15 mL falcon tube and centrifuge the cell suspension for 3 min at 200 × *g* at RT.
  f. Discard the supernatant and gently re-suspend the cell pellet in 1 mL pre-warmed DMEM culturing media.
  g. Count then dilute the cells to a density of 0.5 × 10^5^/mL–1 × 10^5^/mL.
  h. Add 20 ml of cell suspension to each 15 cm dish. With the plate flat on the table gently move each plate horizontally in a figure of 8-shaped manner to distribute the cells evenly across the plate.
  i. Keep the plates in the incubator for 2 days before administering drug treatments.
2. Treat the cells with 4sU+UVA, heat-shock, osmosis or arsenite stresses.
  For the 4sU+UVA treatment, carry out steps a-d.
  a. Add 4sU to the media to a final concentration of 500 μg/mL for 1 h.
    ***Note:*** Check the cells under the microscope before starting the experiments. The cells should be 60% to 70% confluent on the day of stress treatment.
  b. Discard the culturing media, wash the cells once with 6 mL 1xPBS (RT) and then add 10 mL 1xPBS.
  c. Treat the cells with UVA (500 J/m^2^) by the UV crosslinker.
    ***Note:*** The cells should be directly under the UV lamps to receive the correct amount of UV light.
    ***Note:*** The UV does is fixed at 500 J/m^2^, the exposure time may vary depending on the power output of the crosslinker.
  d. Discard the 10 mL 1XPBS, add 20 mL fresh warm DMEM culturing media and put the plates back into the incubator for 8 h.
    For osmosis stress treatment, carry out steps b and d, then e.
  e. Directly add 2.22 ml NaCl stock buffer to the culturing medium to a final concentration of 0.2 M for 1 h.
    For heat-shock stress, carry out steps b and d, then f.
  f. Transfer the dish to the 43°C incubator for 1 h.
    For arsenite stress, carry out steps b and d, then g.
  g. Add arsenite solution to the culturing media to a final concentration of 200 μM and put the plates in the incubator for 0.5 h.
    ***Note:*** Step b and d are applied to all the samples in the same experiment.
    ***Note:*** During the heat-shock, osmosis and arsenite treatment times, prepare fresh B0 buffer, label 1.5 mL DNA low-binding tubes and 15 mL falcon tubes, cool down 1XPBS on ice and the required equipment (swing-out centrifuge, Bioruptor and flow cytometer) to 4°C.
    ***Note:*** During the heat-shock, osmosis and arsenite treatment times, put enough fresh DMEM cell culturing medium (8 mL per sample) in a clean sterile glass bottle and let it warm to RT.

### Cell fixation and sonication

**Timing: 1 h**

3. Cell fixation.
  a. Prepare 80 mL fresh fixation medium by directly adding 5 mL 16% formaldehyde into 75 mL fresh DMEM cell culturing medium (RT).
  b. Discard the culturing medium. Add 8 mL fixation medium (fresh culturing medium with 1% formaldehyde; RT), put the dishes on a rocking shaker, shaking slowly for 15 min at RT.
    ***Note:*** Arsenite- and formaldehyde-containing medium is toxic and should be carefully disposed of by following your local dangerous chemical waste disposal regulations.^7^ Consult with your local Health & Safety department before ordering or performing experiments with arsenite and formaldehyde.
  c. Add 640 μL quench buffer to the dishes, keep them in RT for 5 min on a rocking shaker to stop the fixation process.
  d. Discard the arsenite- and formaldehyde-containing liquid in accordance with appropriate hazardous waste disposal guidelines, wash the cells once with 6 mL cold (4°C) 1× PBS and then add 6 mL cold (4°C) 1× PBS, keep all plates on ice.
    ***Note:*** Step d must be completed for all samples before proceeding to the next steps.
  e. Scrape the cells with a cell scraper gently and transfer the suspension into 15 mL falcon tubes.
  f. Add another 6 mL cold (4°C) 1× PBS to the dishes and scrape the remaining cells and transfer them into the same 15 mL falcon tubes as used in step 3e (the falcon tubes will now have a total volume of approximately 12 mL of cell suspension).
  g. Spin down the cells using a centrifugation speed of 200 × *g* for 5 min at RT. Discard the arsenite and formaldehyde-containing supernatant in accordance with appropriate hazardous waste disposal guidelines. Spin the Falcons again at 200 × *g* for 3 min and discard the remaining PBS by tipping it out with 200 μL tips.
  h. Resuspend the cell pellet in 2.8 mL B0 buffer.
  i. Pipette thoroughly and aliquot 0.7 mL of cell lysate per 1.5 mL DNA low binding tube. The protocol will yield 4 tubes per plate. Keep all cell lysates on ice for 20 min.
    ***Note:*** Keeping the cell lysates on ice for 20 min is very important, this step will significantly prevent sample clog in downstream FACS sorting procedures.
    ***Note:*** In step h, based on our experience, 4sU + UVA samples have fewer cells than other conditions. Therefore, a reduced volume of B0 buffer (∼0.8 fold) should be used to ensure equal cell density in the lysate.
4. Cell lysate sonication.
  a. Use a Bioruptor to sonicate the lysates: 4°C, 2 cycles, on 30 s, off 30 s, low energy.
    ***Note:*** Re-suspend the samples by pipetting up and down several times before sonication. The samples are kept on ice both before and after sonication.
    ***Note:*** Covaris sonicator (Covaris, E220) also works but the set-up (peak power, cycles and duty factor) needs to be optimized. “75 peak power, 200 cycles per burst, 3% duty factor, temperature 4°C” is a good optimization start setting. We have not tested other sonicators.
  b. Merge the same samples in a 5 mL DNA-low binding tube and keep the lysates on ice for 20 min.
  c. Vortex the Falcon/Eppendorf at 20,000 × *g* for 5s.
  d. Filter the cell lysates through a 40 μm filter. Reserve 30 μL of the filtered cell lysates as total cell lysate input for RNA-seq.
    ***Note:*** Although not necessary, pre-wetting the filter with B0 buffer helps the lysates go through the filter membrane.
    ***Note:*** Filtered cell lysate is a better total cell lysate control than unfiltered cell lysate as the filtering process will remove some DNA/protein/RNA debris.

### Stress granule sorting

**Timing: 6 h**

5. Sort the cell lysates with a Symphony S6 or AriaFusion flow cytometer (BD) (Figure 1). Collect the SGs in 1.5 ml DNA-low binding tubes that contain 100 μL B0 buffer and 1 μL RNase inhibitor. See also troubleshooting 1 and 2.

***Note:*** Usually around 400,000 SGs can be collected from the 4sU+UVA-treated samples and five times more from arsenite- or heat-shock-treated samples, which is consistent with the SGs numbers from cell populations.

***Note:*** 7–10 ng RNA can be extracted from 400,000 SGs, which is typically enough for RNA-sequencing after RiboRNA depletion. Discuss with your sequencing facility colleagues or collaborators to decide whether it needs to be scaled up.

***Note:*** Sorting 400,000 SGs from one 4sU+UVA sample (from one 15 cm dish of cells) needs approximately 2.5 h. Collecting 400,000 SGs from 4sU+UVA, arsenite and heat-shock samples needs approximately 2.5, 0.5, and 1 h respectively.

***Note:*** Adding RNase inhibitor to the collection buffer helps protect SGs from probable RNase contamination from the FACS machine or air.

***Note:*** Cool down the FACS machine before sorting to prevent SG RNA degradation.

6. Wash the SGs.
  a. Spin down the SGs by centrifugation at 2000 × *g* for 15 min at 4°C. Discard the supernatant carefully with 200 μL tips, leaving 100 μL buffer in the tube.
    ***Note:*** Use a swing-out rotor to perform the centrifugation so that the pellets (invisible) are enriched at the bases of the tubes.
    ***Note:*** 2000 × g is enough to spin down SGs.
  b. Resuspend the SGs (invisible pellet) in 1 mL cold B0 buffer using 1 mL low retention pipette tips.
  c. spin down the SGs by centrifuging at 2000 × *g* for 15 min at 4°C.
  d. Carefully discard the supernatant using a 1 mL tip, followed by a 200 μL tip for finer removal. Leave approximately 30–50 μL of B0 buffer in the tube to prevent sample loss.
    ***Note:*** Concentrated SGs can be checked under the microscope (Figure 2).
  e. Directly snap-freeze both the SGs samples in step 6b and the input samples in step 4d in liquid nitrogen and store at −80°C.

### RNA extraction from cells and SGs

**Timing: 2.5 h**

7. RNA extraction and library preparation for RNA-sequencing.
  a. Top up the total volume of the isolated SG and input samples to 50 μL with B0 buffer. Digest with 10 μL Proteinase K at 42°C for 40 min in the Thermal mixer at 500 rpm.
  b. De-crosslink by incubation at 65°C for 40 min without shaking.^8^
  c. Extract RNA using the Oligo Clean & Concentrator Kit (Zymo Research, D4060). Use 20 μL of RNase- and DNase-free Water in the last step for RNA elution.
  d. Add 1 μL of Turbo DNase and 2 μL of 10× Turbo DNase buffer to the eluted RNA. Incubate at 37°C for 10 min. Clean up using the Oligo Clean & Concentrator Kit. The RNA is now ready for sequencing. See also troubleshooting 3.

***Note:*** DNase treatment must be applied after de-crosslinking and protein digestion steps to thoroughly remove DNA contamination.

***Note:*** The RNA is fragmented due to crosslinking/sonication/de-crosslinking steps, but it does not interfere with downstream sequencing.

***Note:*** We use the Illumina stranded Total RNA with Ribo-zero Plus protocol for the library preparation.

## Expected outcomes

We anticipate the successful detection and isolation of SGs using flow cytometry-based FANCI and immunofluorescence microscopy. The results should confirm that 4sU+UVA, arsenite, osmosis and heat shock treatment induces SG formation, which can be effectively isolated via FANCI. Immunofluorescence analysis of the isolated SGs is expected to reveal particles with SG-like morphology, closely resembling SGs in the cytosol. These findings would be consistent with our previous publication.^1^

## Limitations

The FANCI protocol can efficiently sort SGs based on G3BP1-mCherry signal, which limits its application to primary or non-tagged cells. Antibody-based FACS and/or FANCI with other endogenous SG markers like CAPRIN1 or USP10 may offer broader applicability in future, but these approaches are still under development and therefore not covered in the current protocol. The current protocol involves formaldehyde fixation which means that isolated SGs cannot be used in biophysical assays or to study SG dynamics. Although our work has demonstrated that FANCI captures both SG cores and some peripheral components—such as P62 (an autophagy receptor associated with 4sU+UVA-induced SGs^1^), we cannot be sure that all dynamic peripheral proteins or RNA species are captured by FANCI.

## Troubleshooting

### Problem 1

Fail to find the SG population when sorting cell lysates (related to Step 5).

### Potential solution

SGs are tiny condensates in the cells and their size is much smaller than intact cells. It is essential to set up the SSC and FSC threshold to 200 (the default value is 5000) to find the SG population. Log Scale of FSC and SSC is needed to identify the SG population in the FACS profile. It is strongly recommended to include a sample from untreated cells as a negative control. The SGs are stable in the cell lysate for 24 h at 4°C but making fresh samples is strongly recommended for RNA sequencing experiments.

### Problem 2

Variation in SG concentration in cell lysates (related to Step 5).

### Potential solution

The efficiency of SG induction varies by stress type. While arsenite induces SGs in more than 80% of the cells, 4sU+UVA induces SGs only in post-mitotic cells.^1^ There is no need to try to normalize the SG concentration in cell lysates. It is strongly recommended to include more than one type of stress in each experiment for better comparison. As SG sorting takes hours for each sample, it is also suggested to prepare one sample from each condition and sort them in the same day and repeat the whole experiment several times in different days to collect enough biological replicates for each condition. It will help exclude batch effects in the downstream analysis although we did not observe any obvious batch effects.

### Problem 3

DNA contamination in recovered RNA (related to Step 7).

### Potential solution

Classical SGs contain only mature mRNA but the non-canonical DHX9 SGs include intron RNA which is indistinguishable from genomic DNA by standard RNA-seq analysis. It is therefore important that DNA contamination is excluded in order to confirm the existence of intron RNA and unspliced pre-mRNA or non-coding RNA.^1^^,^^9^ During the RNA extraction steps (Step 7), the SGs are first digested with Proteinase K to remove proteins and possible contaminating RNases (which are also proteins), then the samples are heated to 65°C for 40 min to de-crosslink protein (remaining peptides) and RNA. The RNA is purified and subjected to DNase treatment. The sequential steps in part 7 of the protocol are important for removing protein and DNA as much as possible and preserving RNA content.

## Resource availability

### Lead contact

Further information and requests for reagents may be directed to and will be fulfilled by the lead contact, Prof. Dr. Asifa Akhtar (akhtar@ie-freiburg.mpg.de).

### Technical contact

Technical questions on executing this protocol should be directed to and will be answered by the technical contact, Dr. Yilong Zhou (zhou@nus.edu.sg).

### Materials availability

Requests for the cell lines generated in this study should be directed to the lead contact and will be fulfilled upon conclusion of the appropriate materials transfer agreement.

### Data and code availability

This study did not generate/analyze any code.

## Acknowledgments

We thank the past and current members of the Akhtar lab for helpful discussions and support. We thank the Flow Cytometry and DNA Sequencing Facilities at MPI Freiburg for their help with experiments, especially Hobitz Sebastian and Schuldes Konrad. This study was supported by the 10.13039/501100001659Deutsche Forschungsgemeinschaft (DFG, 10.13039/501100001659German Research Foundation) under Germany’s Excellence Strategy (CIBSS-EXC-2189-project ID 390939984). This work was also supported by the 10.13039/501100001659DFG under CRC 992 (A02), CRC 1425 (P04), and CRC 1381 (B3), awarded to A.A. Y.Z. was supported by the 10.13039/100004410European Molecular Biology Organization (EMBO) Postdoctoral Fellowship and the 10.13039/100020612National University of Singapore (NUS) Presidential Young Professorship. This work was also supported by the award of the Gottfried Wilhelm Leibniz Prize by the DFG to A.A.

## Author contributions

Y.Z. conceived the project and designed the experiments. Y.Z. performed all the experiments and analyzed the data. A.A. supervised the project. Y.Z. wrote the original manuscript with editing from all other authors. M.S. assisted with experiments and edited the manuscript.

## Declaration of interests

The authors declare no competing interests.
